# Creatine modulates cellular energy metabolism and protects against cancer cachexia-associated muscle wasting

**DOI:** 10.3389/fphar.2022.1086662

**Published:** 2022-12-07

**Authors:** Lulu Wei, Ranran Wang, Kai Lin, Xiaolu Jin, Li Li, Junaid Wazir, Wenyuan Pu, Panpan Lian, Renwei Lu, Shiyu Song, Quan Zhao, Jiabin Li, Hongwei Wang

**Affiliations:** ^1^ State Key Laboratory of Analytical Chemistry for Life Science, Medical School of Nanjing University, Nanjing, China; ^2^ Center for Translational Medicine and Jiangsu Key Laboratory of Molecular Medicine, Medical School of Nanjing University, Nanjing, China; ^3^ Department of Central Laboratory, Yancheng Medical Research Center of Nanjing University Medical School, The First People’s Hospital of Yancheng, Yancheng, China; ^4^ The State Key Laboratory of Pharmaceutical Biotechnology, School of Life Sciences, Nanjing University, Nanjing, China

**Keywords:** cancer cachexia, metabolism, mitochondria, ubiquitination, autophagy

## Abstract

Cancer cachexia is a multifactorial syndrome defined by progressive loss of body weight with specific depletion of skeletal muscle and adipose tissue. Since there are no FDA-approved drugs that are available, nutritional intervention is recommended as a supporting therapy. Creatine supplementation has an ergogenic effect in various types of sports training, but the regulatory effects of creatine supplementation in cancer cachexia remain unknown. In this study, we investigated the impact of creatine supplementation on cachectic weight loss and muscle loss protection in a tumor-bearing cachectic mouse model, and the underlying molecular mechanism of body weight protection was further assessed. We observed decreased serum creatine levels in patients with cancer cachexia, and the creatine content in skeletal muscle was also significantly decreased in cachectic skeletal muscle in the C26 tumor-bearing mouse model. Creatine supplementation protected against cancer cachexia-associated body weight loss and muscle wasting and induced greater improvements in grip strength. Mechanistically, creatine treatment altered the dysfunction and morphological abnormalities of mitochondria, thus protecting against cachectic muscle wasting by inhibiting the abnormal overactivation of the ubiquitin proteasome system (UPS) and autophagic lysosomal system (ALS). In addition, electron microscopy revealed that creatine supplementation alleviated the observed increase in the percentage of damaged mitochondria in C26 mice, indicating that nutritional intervention with creatine supplementation effectively counteracts mitochondrial dysfunction to mitigate muscle loss in cancer cachexia. These results uncover a previously uncharacterized role for creatine in cachectic muscle wasting by modulating cellular energy metabolism to reduce the level of muscle cell atrophy.

## Introduction

Cachexia is a systemic syndrome characterized by involuntary progressive weight loss, mainly affecting skeletal muscle and adipose tissue (DeWys, 1982). A variety of chronic inflammatory diseases, including COPD, heart failure, AIDS, and malignant cancer, can induce the development of cachexia (Baracos et al., 2018). Cancer cachexia occurs in 50%–80% of cancer patients and seriously affects the quality of life and contributes to significant mortality in cancer patients. Currently, there is no effective treatment and no Food and Drug Administration (FDA)-approved drug, that is, available for cancer cachexia; therefore, searching for new therapeutic targets is very important for the establishment of novel treatments for cancer cachexia (Benny Klimek et al., 2010; Zhou et al., 2010).

Although the pathological mechanism of cancer cachexia is not fully understood, it has been well established that the imbalance between anabolic and catabolic metabolism is the major cause for the development of cancer cachexia (Fonseca et al., 2020). Skeletal muscle atrophy is the typical pathological phenotype of cancer cachexia, and metabolic disorders cause the reduced generation of intracellular ATP, which inhibits skeletal muscle cell proliferation and promotes cachexia-associated muscle atrophy (Schiaffino et al., 2013). The development of cachexia-associated muscle wasting is the result of the overactivation of the ubiquitin proteasome system (UPS) and the autophagic lysosomal system (ALS), which are responsible for the degradation of cellular structural proteins and cytoplasmic content, respectively. Within this process, reversing the energy imbalance and increasing muscle cell anabolic metabolism have become major challenges for the prevention and treatment of cachexia-associated muscle wasting (Bodine et al., 2001; Gomes et al., 2001; Lecker et al., 2004; Sacheck et al., 2007; Bonaldo and Sandri, 2013; Chang et al., 2020).

Creatine is a nitrogenous amine that can be used as an alternative energy supplement in tissues with high metabolic demand, such as skeletal muscle and the brain, where most of the body’s creatine is found (Schlattner et al., 2006; Wyss et al., 2007). Creatine supplementation has been widely used in sports to increase muscle strength and for disease treatment. Creatine supplementation has also made advances in the clinical management of neurological and muscle-associated metabolic diseases, such as peripheral neuropathies, intellectual disability, autism, speech delay, myotonic dystrophy, and inflammatory myopathies, where supplementation can provide some relief (Gualano et al., 2012). However, the therapeutic effects of creatine supplementation on cancer cachexia-associated muscle wasting are still not known.

In the current study, we sought to investigate the therapeutic potential of creatine supplementation on cancer cachectic muscle wasting in a cancer cachectic mouse model. The results presented here demonstrate that compared to the untreated cancer cachectic mice, dietary exposure to creatine significantly alleviated cachectic weight loss and protected against skeletal muscle atrophy. These effects were mediated by blocking the abnormal activation of the UPS and ALS, which was accompanied by sustained increased mitochondrial bioenergetics function. Our study provided experimental evidence showing the therapeutic potential of creatine for the treatment of cachexia-associated weight loss and muscle atrophy.

## Materials and methods

### Patients sample

The study was approved by the Ethics Committee of the Medical School of Nanjing University (IRB no: 20200115003). All patients provided written informed consent. Cachexia was defined clinically as documented non-intentional dry weight loss of >5 kg (all >10% of their previous normal weight) at least in ≤3 months (Jatoi et al., 2007). All cachectic patients also complained of weight loss; patients’ skeletal biopsy specimens of abdominal muscles were obtained after hepatobiliary surgery. Control samples were acquired from non-cachectic patients undergoing elective orthopedic surgery.

### Cell culture

The mouse myoblast C2C12 was obtained from the American Type Culture Collection (ATCC, United States) and cultured in Dulbecco’s modified Eagle’s medium (DMEM, BioChannel Biological Technology Co.) supplemented with 10% fetal bovine serum, penicillin (100 mg/ml, BioChannel Biological Technology Co.) and streptomycin (100 mg/ml, BioChannel Biological Technology Co.). To induce myotube formation, cells were grown to 100% confluency and switched to DMEM containing 2% horse serum (HyClone, SH30074.03HI) and 1% penicillin/streptomycin for up to 4–7 days. The mouse tumor cell line colorectal adenocarcinoma colon 26 (C26) was purchased from the American Type Culture Collection (ATCC, United States) and maintained in RPMI 1640 (BioChannel Biological Technology Co.) medium with l-glutamine (Life Technology, New York, NY, United States) containing 10% fetal bovine serum, penicillin (100 mg/ml, BioChannel Biological Technology Co.) and streptomycin (100 mg/ml, BioChannel Biological Technology Co.). The mouse tumor cell line Lewis lung cancer (LLC) was purchased from the American Type Culture Collection (ATCC, United States) and maintained in DMEM (BioChannel Biological Technology Co.) containing 10% fetal bovine serum, penicillin (100 mg/ml, BioChannel Biological Technology Co.) and streptomycin (100 mg/ml, BioChannel Biological Technology Co.). All the cell lines were incubated at 37°C in a humidified chamber with 5% CO_2_.

### Cancer cachexia mouse model

Six-week-old male BALB/c mice and C57BL/6 mice (obtained from the Model Animal Research Centre of Nanjing University) were maintained under specific pathogen-free (SPF) conditions and acclimated for 1 week. Mice were housed in individually ventilated cages (IVCs) with free access to drinking water and a basal diet under controlled humidity, light (12 h light/12 h dark cycle) and temperature conditions. All experiments involving animals were conducted under approved protocols granted by the Institutional Animal Care and Use Committee of Nanjing University. After acclimation, 18 mice were randomized into three groups: normal (NC, *n* = 6), tumor-bearing control (cachexia, *n* = 6) and creatine treatments (cachexia + Cr, *n* = 6). Colon 26 cells (5.0 × 10^5^) in 100 μl phosphate-buffered saline were subcutaneously inoculated into the left flanks of BALB/c mice (cachexia and cachexia + Cr mice) on day 0. The cachexia + Cr group mice received an intraperitoneal injection of 125 mM Cr (dissolved in phosphate-buffered saline, purchased from MCE, HY-W010388, China) every day starting at day 7 after tumor injection. LLC cells (5.0 × 10^5^) in 100 μl phosphate-buffered saline were subcutaneously inoculated into the left flanks of C57BL/6 mice (cachexia and cachexia + Cr mice) on day 0. The cachexia + Cr group mice received an intraperitoneal injection of 125 mM Cr (dissolved in phosphate-buffered saline, purchased from MCE, HY-W010388, China) every day starting on day 7 after tumor injection. When the mice lost 20% of their body weight or when their tumor volumes reached 1500 mm^3^, the mice were sacrificed. Tumors, muscles, and other organs were rapidly dissected, frozen in liquid nitrogen and stored at −80°C until further analysis or fixed with 4% formaldehyde for histological staining.

### Grip strength measurement

A grip strength meter (SA415, Sansbio, Jiangsu, China) was used to assess the forelimb grip strength and four paw grip strength of the mice. Mice were lifted by the tail and induced to grasp rigid grids attached to a digital force gauge. The tail of each mouse was gently pulled backward, and the tension reading of the digital force gauge was defined as the grip strength before the mouse released the net. Five consecutive tests were performed on each mouse, and the mean maximum limb muscle strength value (grams) (g) was calculated.

### Histological analysis

Mice were sacrificed, and tissues were fixed in 4% formaldehyde overnight, followed by dehydration and embedding in paraffin. For histopathological evaluation, 2 µm thick skeletal muscle sections were stained with hematoxylin and eosin (HE) and Periodic Acid–Schiff–diastase (PAS) and examined by light microscopy. Immunofluorescence (IF) staining of paraffin sections was performed using fluorochrome-conjugated antibodies (Laminin, ab11575, Abcam, United Kingdom) following previously reported methods (Niu et al., 2021). Masson trichrome staining was performed with a Masson modified IMEB stain kit (K7298, IMEB Inc. San Marcos, CA) following the protocol. Immunohistochemical (IHC) staining was performed with an immunohistochemistry kit (D601037-0020, Sangon Biotech, China) following the manufacturer’s protocol. The specimens were examined under an FV10i laser scanning confocal microscope (Olympus, Center Valley, PA, United States).

### Immunofluorescence

Cells were plated in 12-well plates coated with poly-d-lysine (0.1 mg/ml, Beyotime, ST508, China) and induced to differentiate. After treatment, the cells were fixed in 4% paraformaldehyde for 30 min, permeabilized with 0.1% Triton X-100 in PBS, and then incubated with anti-myosin heavy chain (MHC) (#MAB4470, R&D Systems) and LC3 (Proteintech, 14600-1-AP, China) (1:100) in 1% BSA/PBST overnight at 4°C. The cells were then incubated with a fluorescence-labeled secondary anti-mouse antibody (1:1000) and DAPI (1:1000) at room temperature for 1 h. The specimens were examined under an FV10i laser scanning confocal microscope (Olympus, Center Valley, PA, United States).

### Western blot

Total cell lysates were solubilized in ice-cold RIPA lysis buffer (Beyotime Biotechnology, P0013B, China) consisting of protease and phosphatase inhibitor cocktails (HY-K0010, HY-K0021, HY-K0022, MCE, United States). The membranes were blocked with 5% bovine albumin for 2 h at room temperature prior to incubation with the indicated primary antibodies. The signals were detected with the following antibodies following standard procedures: phosphorylated STAT3 (CST, #9139), Atrogin-1 (Satan Cruz, sc166806), MuRF-1 (Santa Cruz, sc398608), MHC (R&D Systems, #MAB4470), p62 (CST, #5114s), LC3 (Proteintech, 14600-1-AP), phospho-P70 S6K (Beyotime, AF5899), phospho-EIF4EBP1 (Beyotime, AF5806), phospho-mTOR (Beyotime, AF5869), GDF8/Myostatin (Proteintech, 19142-1-AP), iNOS (Affinity, AF0199), phospho-Akt (Ser473) (CST, #4060), phospho-AMPK (Abcam, ab133448), ATGL (CST, #2439), HSL (CST, #18381), UCP-1 (Satan Cruz, sc518171) and GAPDH (Proteintech, 60004-1-Ig). Subsequently, the membranes were washed and incubated for 2 h at room temperature with peroxidase-conjugated secondary antibodies (Bioworld). Following several washes, chemiluminescent images of immunostained bands on the membranes were recorded on X-ray films using the enhanced chemiluminescence (ECL, FUDE, FD8020, China) system according to the manufacturer’s instructions.

### Real-time PCR assay

Total RNA was extracted from tissues and cell lines using RNA isolator total RNA extraction reagent (Vazyme, R401-01, China), and reverse transcription was performed using PrimeScript RT Master Mix (TaKaRa, Otsu, Japan) and subjected to SYBR Green quantitative real-time PCR using PCR Master Mix (Life Technology) following the manufacturers’ instructions. Q-PCRs were performed on an ABI 7500 real-time PCR system (Applied Biosystems, Waltham, MA, United States). The levels of expression were normalized to the actin levels. The relative expression was calculated using the 2^−ΔΔCT^ method. The specific primer sequences are described in Table 1.

**TABLE 1:** Sequences of primers used in qRT–PCR.

Gene	Forward	Reverse
MuRF-1	5′-ACC​TGC​TGG​TGG​AAA​ACA​TC-3′	5′-AGG​AGC​AAG​TAG​GCA​CCT​CA-3′
Atrogin-1	5′-ATT​CTA​CAC​TGG​CAG​CAG​CA-3′	5′-TCA​GCC​TCT​GCA​TGA​TGT​TC-3′
Slc6a8	5′-GAT​TGC​CCT​GGT​TGG​AGG​AA-3′	5′-GGC​ATA​GCC​CAG​ACC​TTT​GA-3′
Gatm	5′-ACT​AGG​ACC​TTG​TGC​ACG​C-3′	5′-AAG​GAT​CCT​CCA​AGC​CGA​GA-3′
Gamt	5′-TGT​TTG​AGG​AGA​CGC​AGG​TG-3′	5′-AAG​GCA​TAG​TAG​CGG​CAG​TC-3′
CKM	5′-CAA​CAC​CCA​CAA​CAA​GTT​CAA-3′	5′-AGG​TGC​TCG​TTC​CAC​ATG​AA-3′
SIRT1	5′-TGA​TTG​GCA​CCG​ATC​CTC​G-3′	5′-CCA​CAG​CGT​CAT​ATC​ATC​CAG-3′
PGC-1α	5′-AGT​GGT​GTA​GCG​ACC​AAT​CG-3′	5′-GGG​CAA​TCC​GTC​TTC​ATC​CA-3′
SIRT3	5′-GAG​CGG​CCT​CTA​CAG​CAA​C-3′	5′-GGA​AGT​AGT​GAG​TGA​CAT​TGG​G-3′
PPARd	5′-TCC​ATC​GTC​AAC​AAA​GAC​GGG-3′	5′-ACT​TGG​GCT​CAA​TGA​TGT​CAC-3′
Nrf1	5′-AGC​ACG​GAG​TGA​CCC​AAA​C-3′	5′-AGG​ATG​TCC​GAG​TCA​TCA​TAA​GA-3′
PGC-1β	5′-CTT​GGC​TGC​GCT​TAC​GAA​GA-3′	5′-GAA​AGC​TCG​TCC​ACG​TCA​GAC-3′
TFAM	5′-ATT​CCG​AAG​TGT​TTT​TCC​AGC​A-3′	5′-TCT​GAA​AGT​TTT​GCA​TCT​GGG​T-3′
Dnm1L	5′-CCT​CAG​ATC​GTC​GTA​GTG​GGA-3′	5′-GTT​CCT​CTG​GGA​AGA​AGG​TCC-3′
Fis1	5′-TGT​CCA​AGA​GCA​CGC​AAT​TTG-3′	5′-CCT​CGC​ACA​TAC​TTT​AGA​GCC​TT-3′
Mff	5′-ATG​CCA​GTG​TGA​TAA​TGC​AAG​T-3′	5′-CTC​GGC​TCT​CTT​CGC​TTT​G-3′
Mief1	5′-GGT​GAG​CGC​AAA​GGG​AAG​AA-3′	5′-AAT​GCC​CAA​CAT​AGC​TGC​TCC-3′
Mfn1	5′-CCT​ACT​GCT​CCT​TCT​AAC​CCA-3′	5′-AGG​GAC​GCC​AAT​CCT​GTG​A-3′
Mfn2	5′-AGA​ACT​GGA​CCC​GGT​TAC​CA-3′	5′-CAC​TTC​GCT​GAT​ACC​CCT​GA-3′
Opa1	5′-TGG​AAA​ATG​GTT​CGA​GAG​TCA​G-3′	5′-CAT​TCC​GTC​TCT​AGG​TTA​AAG​CG-3′
Oma1	5′-TCT​CTG​GAG​TGA​ATA​ACC​TGG​C-3′	5′-GCA​CTT​GAG​AGG​CAT​CTT​GAT​T-3′
NDUFA9	5′-GTC​CGC​TTT​CGG​GTT​GTT​AGA-3′	5′-CCT​CCT​TTC​CCG​TGA​GGT​A-3′
NDUFS3	5′-TGG​CAG​CAC​GTA​AGA​AGG​G-3′	5′-CTT​GGG​TAA​GAT​TTC​AGC​CAC​AT-3′
SDHA	5′-GGA​ACA​CTC​CAA​AAA​CAG​ACC​T-3′	5′-CCA​CCA​CTG​GGT​ATT​GAG​TAG​AA-3′
UQCRFS1	5′-GAG​CCA​CCT​GTT​CTG​GAT​GTG-3′	5′-GCA​CGA​CGA​TAG​TCA​GAG​AAG​TC-3′
ATP5A	5′-TCT​CCA​TGC​CTC​TAA​CAC​TCG-3′	5′-CCA​GGT​CAA​CAG​ACG​TGT​CAG-3′
Actin	5′-CTA​AGG​CCA​ACC​GTG​AAA​AG-3′	5′-ACC​AGA​GGC​ATA​CAG​GGA​CA-3′

### Creatine assay

The creatine content was measured using a Creatine Assay Kit (Colorimetric/Fluorometric) (Abcam, ab65339) according to the manufacturer’s instructions. The detection was performed using a spectrophotometer (Thermo) at 570 nm.

### COX activity assay

Mitochondrial respiratory chain complex activities were measured by assay kits according to the manufacturer’s protocol (Micro Mitochondrial Respiratory Chain Complex I Activity Assay Kit, BC0515; Micro Mitochondrial Respiratory Chain Complex II Activity Assay Kit, BC3230; Micro Mitochondrial Respiratory Chain Complex III Activity Assay Kit, BC3245; Micro Mitochondrial Respiratory Chain Complex IV Activity Assay Kit, BC0945, Solarbio, Beijing, China).

### Transmission electron microscopy (TEM)

The cultured cells were digested with trypsin and centrifuged (3000 rpm, 2 min), the supernatant was discarded, 2.5% room temperature glutaraldehyde fixative was added, and the cell mass was gently picked up and suspended in the fixative and fixed in the dark at room temperature for 30 min. The cells were dehydrated in 70%, 90% and 100% alcohol sequentially. After infiltration embedding, the cells were cut into ultrathin sections of 60–80 nm. The sections were double stained with uranium–lead (2% uranyl acetate saturated aqueous solution, lead citrate, each for 15 min) and dried overnight at room temperature. The mitochondria were photographed using a HITACHI HT 7800 120 kV microscope.

### Statistical analysis

The data were analyzed by SPSS 18.0 statistical software. The measured data are expressed as the mean ± standard deviation (mean ± SD). The measurement data were tested by Student’s T test and repeated-measurement analysis of variance (ANOVA). Assuming that the test level was determined by α = 0.05, *p* < 0.05 was considered statistically significant. The graph was drawn using GraphPad 7.0 software.

## Results

### Protective effect of creatine supplementation on experimental cancer cachexia

**FIGURE 1:**
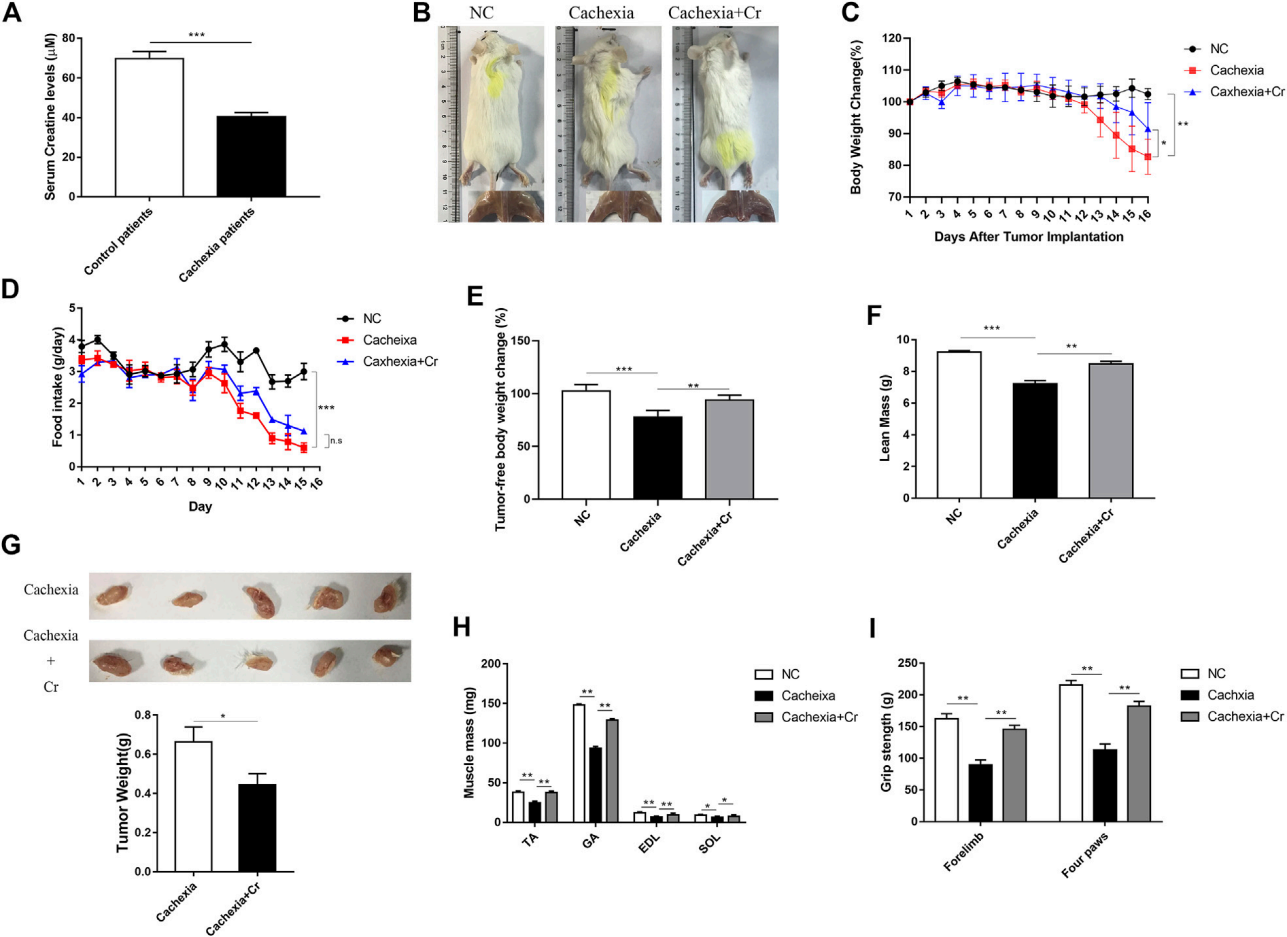
Creatine supplementation protects against experimental cancer cachexia. **(A)** Measurement of creatine levels in the serum from non-cachectic patients and cachexia patients. ***p < 0.0001 denoted non-cachectic patients and cachexia patients. **(B)** Representative general images and skeletal muscle images of the three groups. NC group: normal control mice, cachexia group: C26 tumor-bearing mice, cachexia + Cr group: Creatine-treated C26 tumor-bearing mice. **(C)** Body weight changes in the three groups (mean ± SD, *n* = 6). Day 0: Tumor implantation. Day 7: Creatine injection began. Day 15: Terminal day. **p < 0.01 denoted the NC group versus the cachexia group, *p < 0.05 denoted the cachexia group versus the cachexia + Cr group. **(D)** Daily food consumption in the three groups during the course of the experiment. (mean ± SD, *n* = 6). ***p < 0.001 denoted the NC group versus the cachexia group. There was no significant difference between the cachexia group and the cachexia + Cr group. **(E)** Tumor removal weight changes in the three groups (mean ± SD, *n* = 6). ***p < 0.001 denoted the NC group versus the cachexia group, **p < 0.01 denoted the cachexia group versus the cachexia + Cr group. **(F)** The lean mass changes in the three groups (mean ± SD, *n* = 6). ***p < 0.001 denoted the NC group versus the cachexia group, **p < 0.01 denoted the cachexia group versus the cachexia + Cr group. **(G)** The tumor weight changes in the cachexia group and the cachexia + Cr group (mean ± SD, *n* = 6). *p < 0.05 denoted the cachexia group versus the cachexia + Cr group. **(H)** Skeletal muscle masses of tibialis anterior (TA), gastrocnemius (GA), extensor digitorum longus (EDL) and soleus (SOL). Data are shown as the mean ± SD, *n* = 6, **p < 0.01, *p < 0.05. **(I)** Skeletal muscle strengths were determined based on the grip strength of the forelimbs and four paws. Data are shown as the mean ± SD, *n* = 6, **p < 0.01.

We first measured the serum creatine content of the clinical cancer cachexia patients and found that the average creatine level was significantly decreased in cancer cachexia patients compared with non-cachectic patients (control group) (Figure 1A). To verify that creatine might participate in skeletal muscle energy metabolism regulation in cancer cachexia, we investigated the impact of creatine supplementation on the pathological development of cancer cachexia in a C26 tumor-bearing cachectic mouse model. As shown in Figure 1B, we observed obvious cachectic protective effects of creatine. The physical characteristics of the cachexia mice had a drier and leaner morphology and dull and disheveled fur, and creatine treatment significantly improved their physical condition. Additionally, there was significant body weight loss in the cachexia mice, while creatine supplementation partially alleviated the cachectic weight loss (Figure 1C). We noticed that the protective effects of creatine on cachectic body weight loss were not due to the amelioration of appetite, since creatine supplementation had no effect on the amount of food consumed by mice (Figure 1D). Measurement of the tumor-free weight showed that creatine supplementation significantly increased the tumor-free body weight (Figure 1E). Consistently, creatine treatment also significantly increased lean mass, which is made up primarily of skeletal muscle and bone, in cancer cachexia mice (Figure 1F). Moreover, we also observed that creatine treatment also reduced the tumor weight, suggesting that creatine may have an inhibitory effect on tumor growth (Figure 1G). Additionally, we determined the muscle mass of the tibialis anterior (TA), gastrocnemius (GA), extensor digitorum longus (EDL), and soleus (SOL) in the three groups. We found that creatine treatment protected skeletal muscle from atrophy in these four muscle groups (Figure 1H). To further explore changes in skeletal muscle function, we performed a grip strength test, and the results showed that creatine treatment markedly increased the grip strength of the forelimb and four paws (Figure 1I). At the same time, we used another mouse model of cachexia to observe the phenotypic changes due to creatine injection. We found that creatine protected muscle and lipid tissue from atrophy (Supplementary Figure S1). Together, our results confirm that creatine could protect against tumor-induced cachectic weight loss in both BALB/c mice and C57BL/6 mice.

### Creatine supplementation protects against skeletal muscle atrophy by inhibiting activation of the UPS and ALS

**FIGURE 2:**
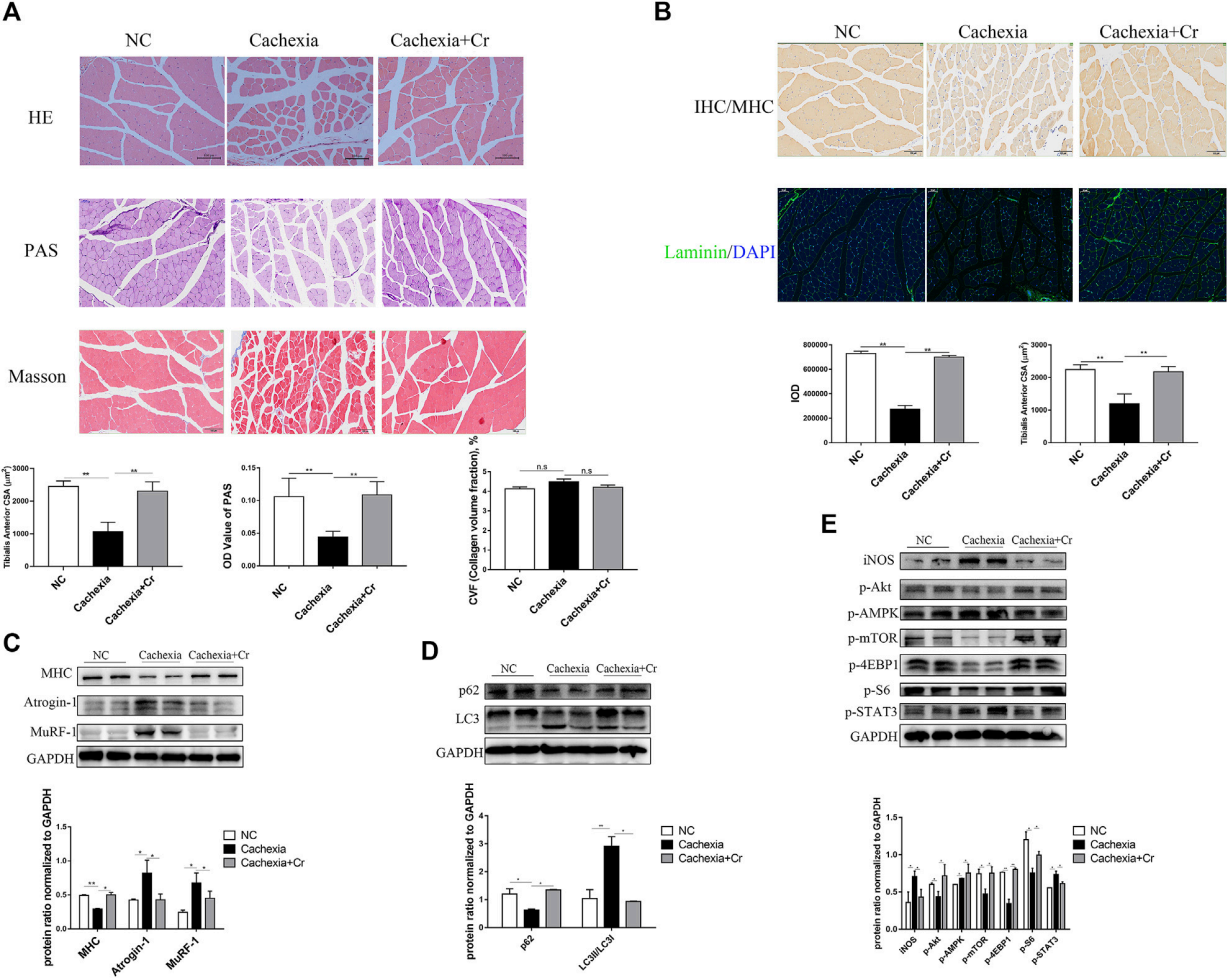
Supplementation with creatine inhibits UPS and ALS activation, thereby preventing skeletal muscle atrophy. **(A)** Representative HE staining showing the morphological changes in the muscles of the three groups. Average size of muscle fiber cross-sectional area in the NC mice, cachectic mice, or creatine-treated cachectic mice. Representative Masson’s trichrome staining of paraffin sections from muscle fibers of different groups of mice is shown. The bar graph shows the collagen volume fraction (CVF) measured by ImageJ. The glycogen content was determined by Periodic Acid-Schiff (PAS) staining. The histogram shows the optical density (OD) of PAS staining. **(B)** Gastrocnemius myosin heavy chain (MHC) expression was evaluated by immunofluorescence (IF) staining. The bar graph shows the mean gray value of different groups. Gastrocnemius sections were stained with an antibody against laminin and DAPI. The bar graph shows the mean gray value of different groups. **(C)** Western blot analysis was used to evaluate MHC, Atrogin-1 and MuRF-1 expression in the three groups (mean ± SD, *n* = 6). *p < 0.05, **p < 0.01. **(D).** Western blot analysis was used to evaluate p62 and LC3 expression in the three groups (mean ± SD, *n* = 6). *p < 0.05, **p < 0.01. **(E)** Western blot analysis was used to evaluate iNOS, p-Akt, p-AMPK, p-mTOR, p-4EBP1, p-S6 and p-STAT3 in the three groups (mean ± SD, *n* = 6). *p < 0.05, **p < 0.01. Data are expressed as the mean ± SD.

The above results suggest that creatine supplementation has a protective effect on the body weight of cancer cachexia mice, mainly through the amelioration of cachectic skeletal muscle wasting. To verify this effect, we performed histopathological analysis, including HE staining, PAS staining, Masson staining, IF staining, and IHC staining, to investigate the atrophy status of skeletal muscle fibers. HE staining of skeletal muscles revealed that creatine supplementation increased the fiber cross-sectional area, which was significantly decreased in cachectic skeletal muscle from C26 tumor-bearing mice (Figures 2A,B). Muscle morphology is closely related to fiber type shifts. Using myosin heavy chain (MHC) IHC staining, we found that creatine supplementation significantly upregulated slow-twitch fibers (Figure 2B). IF staining of laminin showed an increased number of fibers containing centrally located nuclei in cancer cachectic skeletal muscle, suggesting degeneration-regeneration processes in cachectic skeletal muscles, which was reversed by creatine supplementation (Figure 2B). The Masson staining experiment showed that no obvious fibrotic lesions occurred in cancer cachexia, and creatine supplementation did not significantly alter the content of collagen fibers. Glycogen content, as an important substrate of energy metabolism in skeletal muscle, decreased in cachexia mice. Supplementation with creatine increased muscular glycogen in cachexia mice (Figure 2A). To further address the molecular mechanisms underlying the protective effect of creatine on skeletal muscle atrophy, we next measured the activation of the UPS and ALS, the two key signaling pathways involved in the regulation of skeletal muscle atrophy degradation. We observed that creatine supplementation significantly inhibited the expression of the E3 ubiquitin ligases MuRF-1 and Atrogin-1 and restored the expression of MHC during cachectic skeletal muscle atrophy (Figure 2C). There was increased expression of p62 and decreased expression of LC3I/LC3II in cancer cachexia skeletal muscle, suggesting the activation of autophagy in cachexia skeletal muscle. Creatine supplementation reversed these effects, indicating that supplementation with creatine could reduce autophagic flux in skeletal muscle atrophy (Figure 2D).

Cancer cachexia is associated with energy deficiency, so we next examined the activation of proteins related to the PI3K/Akt signaling pathway. As shown in Figure 2E, there was reduced activation of Akt, mTOR and its downstream indicators 4EBP1 and S6. The increased phosphorylation of AMPK in cancer cachexia skeletal muscle suggested an inadequate energy supply-induced cell growth arrest (Figure 2E), and supplementation with creatine reversed the abnormalities of these protein changes. In addition, the increased expression of iNOS in skeletal muscle during cancer cachexia suggests the presence of oxidative stress, which can be alleviated by supplementation with creatine. STAT3, a transcription factor known to be important in cancer cachexia, has been shown to be closely associated with abnormal activation of STAT3 in cancer cachexia, and supplementation with creatine also inhibited its phosphorylation (Figure 2E).

### Creatine supplementation protects adipose tissue by inhibiting white fat browning

**FIGURE 3:**
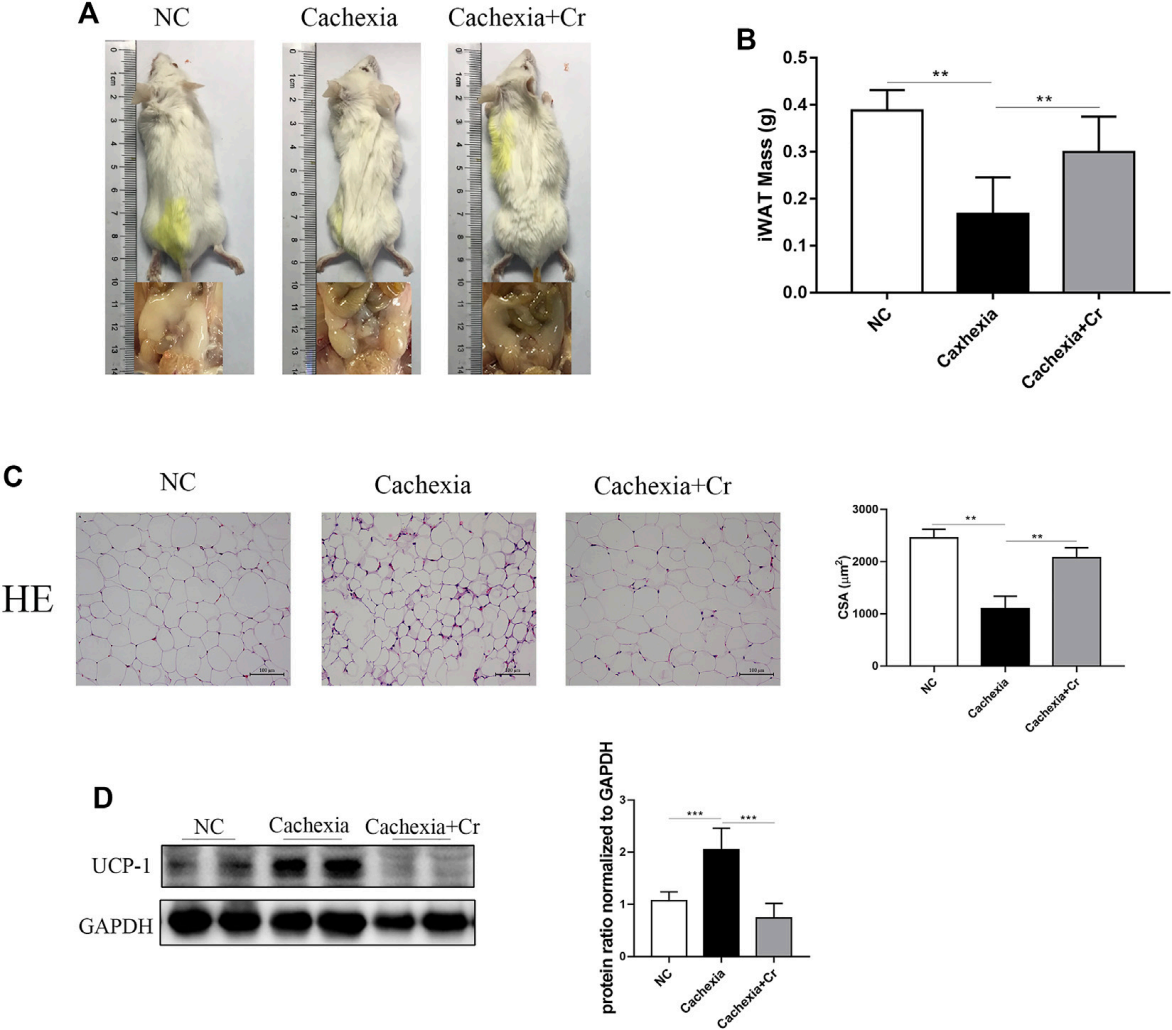
Creatine prevents the browning of white adipose tissue, thereby protecting against adipose tissue wasting. **(A)** Representative general images and homologous groin images of the three groups. NC group: normal control mice, cachexia group: C26 tumor-bearing mice, cachexia + Cr group: Creatine-treated C26 tumor-bearing mice. **(B)** iWAT mass changes in the three groups (mean ± SD, *n* = 6). **p < 0.01 denoted the NC group versus the cachexia group, **p < 0.01 denoted the cachexia group versus the cachexia + Cr group. **(C)** Representative HE staining showing the morphological changes in the adipose tissue of the three groups. Average size of adipose tissue cross-sectional area in the three groups. **(D)** Western blot analysis was used to evaluate UCP-1 expression in the three groups (mean ± SD, *n* = 6). *p < 0.05, **p < 0.01, ***p < 0.001. Data are expressed as the mean ± SD.

In our *in vivo* study, we noticed that cachectic mice and mice given creatine had different body weight changes. To test the impact of creatine supplementation on adipocytes, we next analyzed the fat mass storage in different groups. As expected, compared to healthy control mice, cachectic mice developed obvious atrophy of inguinal fat, but creatine treatment significantly reduced the cachectic adipocyte atrophy level. Mice in the cachexia + Cr group had improved morphology, and inguinal fat was visible (Figure 3A). The inguinal fat of the mice was removed and weighed, and the iWAT weight of the mice in the NC group was significantly decreased compared to that of the mice in the cachexia group. The iWAT weight of the mice in the cachexia + Cr group was significantly increased compared to that of the mice in the cachexia group, indicating that creatine supplementation had a protective effect on the loss of white fat caused by cancer cachexia (Figure 3B). HE staining analysis revealed that the lipid droplets in the adipose tissue of cachexia group mice were significantly smaller than those in the NC group mice. In comparison to the cachexia group, mice in the cachexia + Cr group had significantly larger lipid droplets in the adipose tissue (Figure 3C). In addition, Western blot analysis demonstrated that in adipose tissue, the expression of UCP-1, the key rate-limiting enzyme in fat degradation, was significantly increased in the adipose tissue of mice in the cachexia group. Creatine treatment reversed this effect, suggesting that the adipose tissue of mice in the cachexia group was browned and used more energy for thermogenesis, which was inhibited by creatine supplementation (Figure 3D). These findings indicate that creatine protects against adipose tissue wasting by inhibiting the browning of white adipose tissue.

### Creatine supplementation corrects abnormalities in creatine metabolism in cachectic mice

**FIGURE 4:**
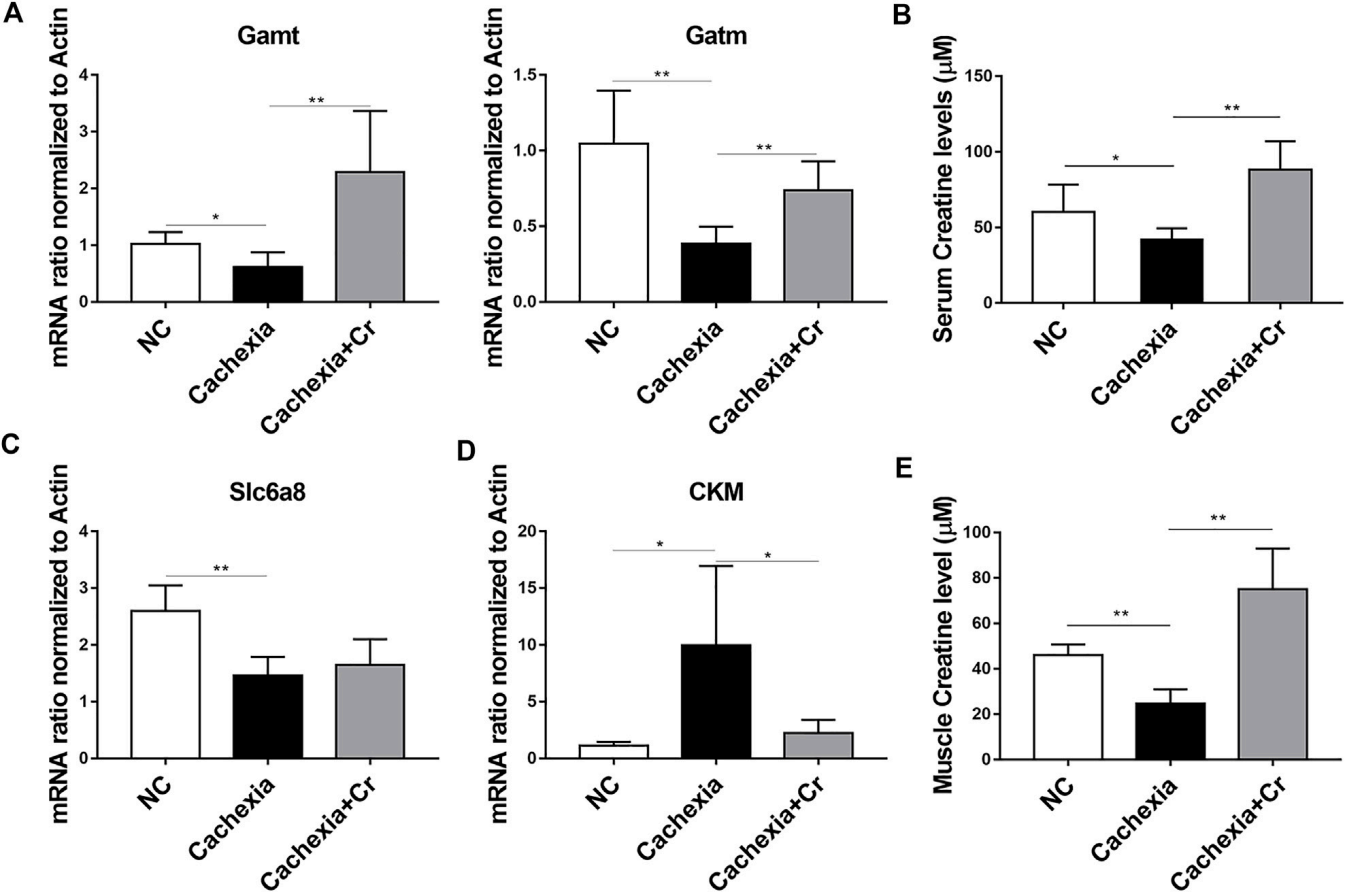
Creatine supplementation corrects abnormal creatine metabolism in cachectic mice. **(A)** Total RNA was extracted from the livers of the three groups, and the expression levels of Gamt and Gatm were measured by real-time PCR. *p < 0.05, **p < 0.01. **(B)** Measurement of creatine levels in the serum from the NC group, cachexia group and cachexia + Cr group. *p < 0.05 denoted the cachexia group versus the cachexia + Cr group. There was no significant difference between the NC group and the cachexia group. **(C,D)** Total RNA was extracted from the gastrocnemius of the three groups, and the expression levels of Slc6a8 and CKM in the gastrocnemius were measured by real-time PCR. *p < 0.05, **p < 0.01. **(E)** Measurement of creatine levels in the muscle from the NC group, cachexia group and cachexia + Cr group. **p < 0.01 denoted the NC group versus the cachexia group. **p < 0.01 denoted the cachexia group versus the cachexia + Cr group. Data are expressed as the mean ± SD.

To address the molecular mechanism of creatine-induced weight loss protection in a cachectic mouse model, we next checked the expression of the enzymes involved in the regulation of creatine metabolism**.** The qPCR results showed that the mRNA expression levels of Gamt and Gatm, two key rate-limiting enzymes for creatine synthesis, were decreased in liver tissues of mice in the cachexia group and increased in liver tissues of mice in the cachexia + Cr group compared with the NC group, suggesting that creatine supplementation could restore the impaired creatine synthesis capacity in the liver (Figure 4A). Compared with the NC group, serum creatine levels in mice in the cachexia group were significantly decreased, and intraperitoneal injection of creatine significantly increased serum creatine levels compared with the cachexia group (Figure 4B). We also examined the mRNA expression levels of the creatine transporter protein Slc6a8. The results of the qPCR analysis revealed that Slc6a8 mRNA expression levels were significantly lower in the skeletal muscle of mice in the cachexia group than in the NC group and that supplementation with creatine had no effect on Slc6a8 mRNA expression levels (Figure 4C). In addition to the ability to translocate creatine from extracellular to intracellular, we also examined the mRNA expression levels of creatine kinase (CKM) in skeletal muscle. Creatine is phosphorylated to form phosphocreatine in the presence of CKM, resulting in the production of large amounts of ATP, and it has been shown that CKM is compensatorily upregulated in the presence of impaired cellular energy status (Jiang et al., 2021). The qPCR results showed that, in comparison to the NC group, the expression level of CKM mRNA was significantly increased in the skeletal muscle of mice in the cachexia group and decreased following creatine supplementation (Figure 4D). In humans, 95% of creatine is stored in skeletal muscle, so we examined the creatine content in skeletal muscle, and the results showed that the creatine content of skeletal muscle was significantly decreased in cachectic mice compared to the NC group and that creatine supplementation significantly increased the creatine content of skeletal muscle (Figure 4E). The above results indicate that creatine supplementation can improve the creatine synthesis capacity in liver tissue and creatine content in skeletal muscle and serum, has no significant improvement in the impaired creatine transport capacity in skeletal muscle, and can inhibit the abnormally elevated CKM mRNA level. Creatine supplementation increases the creatine content in skeletal muscle, provides ATP for the physiological activities of skeletal muscle cells, and protects skeletal muscle from degradation.

### Creatine supplementation prevents myotubular atrophy by inhibiting activation of the UPS and ALS

**FIGURE 5:**
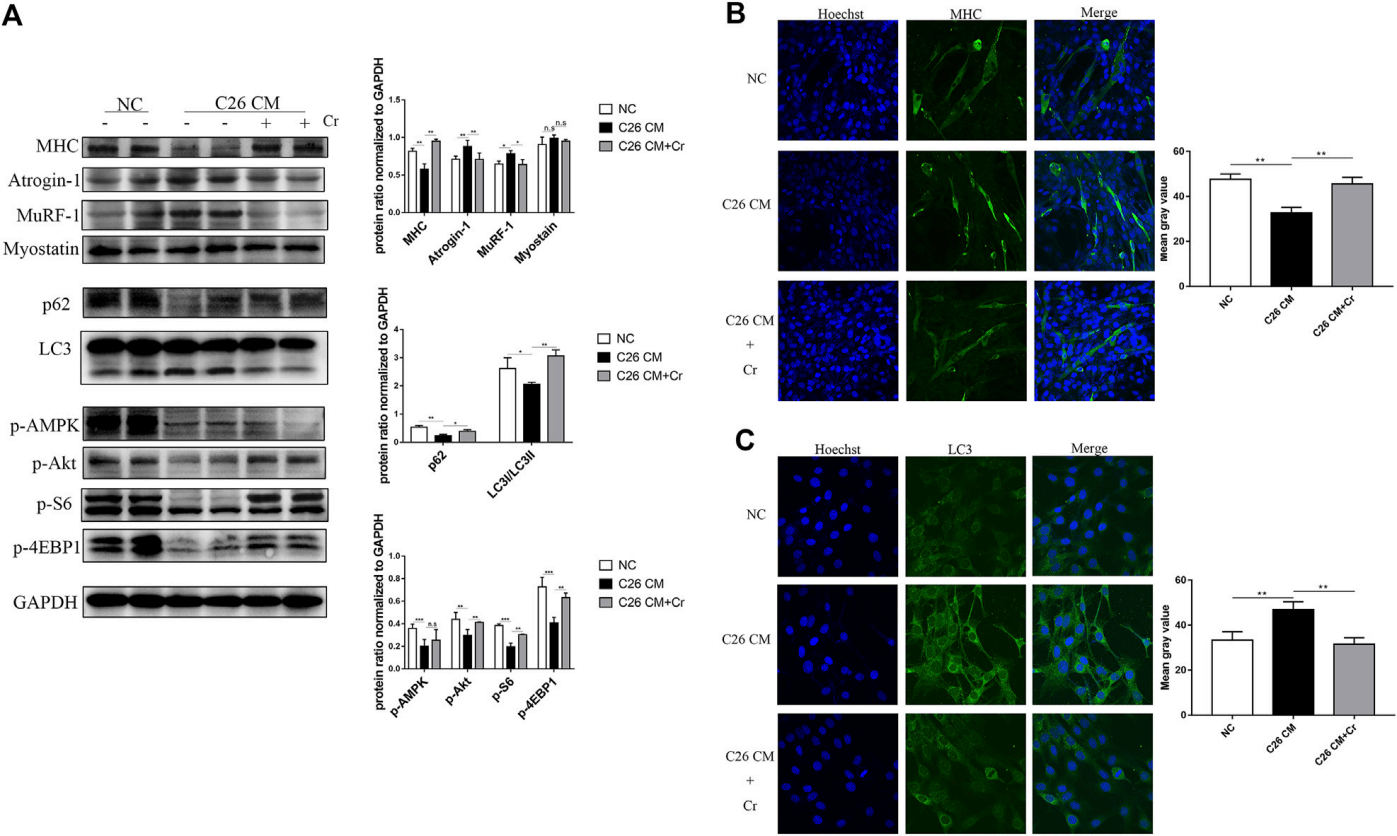
Supplementation with creatine prevents myotubular atrophy by inhibiting the activation of UPS and ALS. **(A)** Western blot analysis was performed to evaluate the expression of MHC, Atrogin-1, MuRF-1, Myostatin, p62, LC3, p-AMPK, p-Akt, p-S6 and p-4EBP1 in the NC group, cachexia group and cachexia + Cr group (mean ± SD, *n* = 6). *p < 0.05, **p < 0.01, ***p < 0.001. **(B)** Myosin heavy chain (MHC) expression in the cell model was evaluated by IF staining. MHC, green, DAPI, blue. The bar graph shows the mean gray value of different groups. **(C)** LC3 expression in the cell model was evaluated by IF staining. LC3, green, DAPI, blue. The bar graph shows the mean gray value of different groups. Data are expressed as the mean ± SD, **p < 0.01.

To investigate the molecular mechanism of creatine inhibition of skeletal muscle degradation, we used the culture medium supernatant of the mouse colorectal cancer cell line C26 to induce a mouse C2C12 myoblast as an *in vitro* cachectic cell model. Western blot analysis revealed that when C2C12 cells were cocultured with C26 supernatant (C26 CM group), the expression of the E3 ubiquitin ligases MuRF-1 and Atrogin-1 was significantly increased, the expression of MHC was decreased, and the addition of creatine reversed this change, indicating that creatine could inhibit myotubular atrophy induced by the UPS (Figure 5A). In the C26 CM group, p62 expression was increased, and LC3I/LC3II expression was decreased, suggesting that autophagy occurred, and the addition of creatine decreased p62 expression and increased LC3I/LC3II expression, indicating a decrease in autophagic flux. Consistent with the *in vivo* data, the expression of p-Akt, p-S6, and p-4EBP1 decreased in the C26 CM group, suggesting an intracellular energy deficiency, and the addition of creatine restored the expression of these proteins, indicating an improvement in the energy deficiency state (Figure 5A). We performed IF experiments on the cells to visually demonstrate this result. MHC IF results indicated that myotubes exhibited significant atrophy in the C26 CM group, but creatine supplementation prevented this pathological change (Figure 5B). In addition, the IF results of LC3 showed that there was significant punctate accumulation in the cells of the C26 CM group, indicating the generation of autophagy, and the punctate accumulation was reduced after supplementation with creatine, indicating that supplementation with creatine could inhibit the abnormal activation of autophagy induced by C26 supernatant (Figure 5C). The above results suggest that creatine can inhibit the activation of the UPS and ALS to protect myotubes from atrophic degradation in an *in vitro* cell model.

### Creatine supplementation improves mitochondrial function and morphology

**FIGURE 6:**
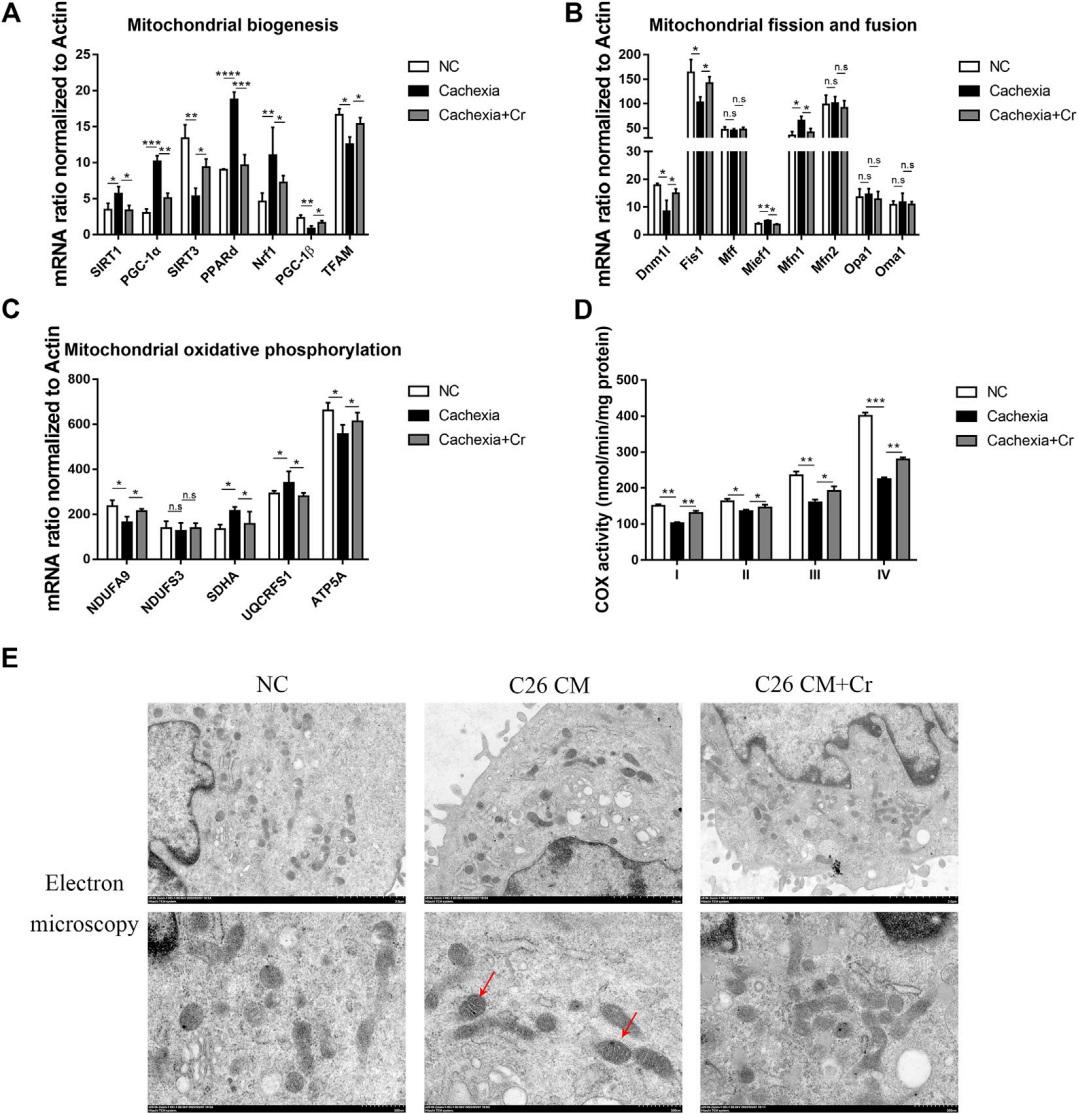
Supplementation with creatine improves mitochondrial function and morphology. **(A–C)** Total RNA was extracted from the skeletal muscle of the NC group, cachexia group and cachexia + Cr group, and the expression levels of genes involved in mitochondrial biogenesis, mitochondrial fission and fusion, and mitochondrial oxidative phosphorylation in muscle were measured by real-time PCR. Data are expressed as the mean ± SD, *p < 0.05, **p < 0.01, ***p < 0.001, ****p < 0.0001, *n* = 6. **(D)** Activities of mitochondrial complexes I-IV in the muscle of the three mouse groups. **(E)** Images of mitochondrial structure were taken by transmission electron microscopy. The red arrows point to structurally abnormal mitochondria.

Mitochondrial dysfunction has been considered a cause of energy failure and induces protein catabolism in cancer cachexia. To assess whether creatine supplementation could influence mitochondrial function, we next analyzed the expression of mitochondrial biogenesis genes. SIRT1 and SIRT3 are two major members of the mammalian sirtuin family of proteins that depend on NAD + as a coenzyme to exert deacetylase activity. We found increased expression of SIRT1 and decreased expression of SIRT3 (Figure 6A), indicating reduced energy generation and mitochondrial dysfunction in cachectic skeletal muscle. PGC-1α is a nuclear transcriptional coactivator that plays an important role in mitochondrial biogenesis and is sensitive to intracellular ATP levels. Its expression, together with the expression of the PGC-1α coactivators PPARδ and Nrf1, was also significantly increased. In contrast to the changes in PGC-1α, another member of the Ppargc family, PGC-1β, and mitochondrial transcription factor A (TFAM), reduced mRNA expression in cachectic skeletal muscle, inducing abnormal mitochondrial structure and function, whereas the application of creatine reversed the above changes (Figure 6A).

Mitochondrial fission and fusion are necessary to maintain stable mtDNA and muscle function. qPCR results revealed that the mRNA levels of genes involved in mitochondrial fission were significantly altered in cachectic mouse skeletal muscle. Mitochondrial fusion proteins 1 and 2 (Mfn1, Mfn2) regulate outer mitochondrial membrane fusion, and optic atrophy type 1 (OPA1) and OMA1 zinc metallopeptidase (Oma1) mediate inner mitochondrial membrane fusion. Our q-PCR data showed that the mRNA expression levels of Mfn1 in the skeletal muscle of cachectic mice were significantly increased, with no difference in the mRNA expression levels of other genes, and this effect was reversed in creatine-treated mice. These findings suggest that cachexia disrupts the dynamic balance of mitochondrial fission and fusion in skeletal muscle and that creatine supplementation can restore mitochondrial homeostasis (Figure 6B).

Cancer cachexia skeletal muscle exhibits low expression of OXPHOS subunit proteins (complex III, IV, and V) (Kitaoka et al., 2021). Our study confirmed this finding, as the genes encoding different subunits of the mitochondrial complex, including NDUFA9 (encoding mitochondrial complex I) and ATP5A (encoding mitochondrial complex V), were significantly downregulated in cachectic muscle. Creatine treatment prevented declines in the levels of oxidative phosphorylation proteins and activities of citrate synthase and cytochrome c oxidase in the skeletal muscle of C26 mice (Figure 6C).

A key component of energy metabolism is respiration, which involves four respiratory chain protein complexes (I-IV) located on the inner membrane of mitochondria (Zhou et al., 2020). Therefore, we analyzed the activity of the mitochondrial complex (I-IV). Complex I-IV activity was significantly decreased in cachectic muscle, and creatine treatment significantly increased complex I-IV activity (Figure 6D).

Finally, we analyzed the mitochondrial morphology. Mitochondria from the skeletal muscle cells in the NC group were full in shape, with intact outer membranes and ridges in the shape of septa, clearly arranged and perpendicular to the long axis of mitochondria. However, in the C26 cachectic group, the number of mitochondria in skeletal muscle was reduced, some mitochondria were swollen, the orientation of mitochondrial cristae was disturbed, blurred, or dissolved, some mitochondrial cristae were cavitated, and cristae gaps were dilated. In response to creatine treatment, the mitochondrial structure was intact, the reduced number was recovered, the mitochondrial cristae were clearly arranged, and there was no evidence of vacuole formation (Figure 6E). The mitochondrial matrix and mitochondrial cristae are the primary sites of energy metabolism in mitochondria, and disruption of the mitochondrial structure directly impacts mitochondrial function. Treatment with creatine significantly increased activity in cachectic mice compared with untreated mice.

## Discussion

Cancer cachexia is a multifactorial syndrome, that is, highly prevalent in metastatic cancers such as pancreatic, gastric, lung, and colorectal cancers (Fearon et al., 2011). Cancer cachexia is driven by altered energy metabolism, including increased energy expenditure, increased catabolism, and inflammatory response, and unlike starvation and simple malnutrition, additional nutritional supplementation does not alleviate the formation and progression of cancer cachexia (Fearon et al., 2011), and there are no FDA-approved drugs that are effective for the treatment of cancer cachexia. Previous studies have shown the potential role of a number of drug candidates for the treatment of cachectic skeletal muscle wasting, including androgens, selective androgen receptor modulators, anti-myostatin drugs, growth hormone and insulin-like growth factor; however, clinical data suggest that these drugs are not sufficiently effective (Anker and Coats, 2002; Jatoi et al., 2007). The most effective clinical treatment for cancer cachexia remains the treatment of the primary disease, supplemented by symptomatic therapy, but cancer cachexia is commonly seen in patients with intermediate to advanced cancer, and the means to treat the primary disease are limited (Morley et al., 2006).

In patients with cancer cachexia, conventional nutritional interventions with common substrates of energy metabolism, such as sugar and fat, do not provide much improvement in reducing cachexia symptoms; moreover, although adipose tissue is also important, excessive obesity does not provide a survival advantage to patients with cachexia (Prado et al., 2020). Therefore, the metabolic pattern of cancer cachexia belongs to nutrition support responsiveness rather than malnutrition. In addition to conventional nutritional interventions, other nutritional intervention approaches will be necessary for the treatment of cancer cachexia.

Creatine is a nitrogenous organic acid with the chemical formula C 4H9 N3 O2, that is, naturally found in vertebrates and assists in providing energy to muscle and nerve cells. Michel Eugène Chevreul first discovered creatine in skeletal muscle in 1832, and later, after the Greek word “Kreas” (meat), named it “Creatine” (Chevreul, 1835). Unlike sugar and fat, creatine can produce ATP rapidly in the presence of creatine kinase to provide energy to skeletal muscle. Creatine is synthesized endogenously, with the vast majority found in skeletal muscle and approximately 66% stored as phosphocreatine. Creatine has been shown to increase energy availability during strenuous exercise, improve recovery from muscle fatigue, and increase muscle strength (Sakkas et al., 2009). However, the functional role of creatine in cancer cachexia still needs to be defined.

Previous studies indicated that the level of serum creatine correlated with the development of cancer cachexia. In late-stage cancer patients, such as non-small cell lung cancer (NSCLC), low serum creatinine levels were associated with a lower muscle cross-sectional area compared to patients with normal serum creatinine levels, suggesting that serum creatinine levels might be an important biomarker of cancer cachexia-associated muscle wasting (Jiang et al., 2021; Li et al., 2021). In our study, we observed a decreased serum level of creatine in cachectic patients, and in the skeletal muscle of cachectic mice, the creatine content was significantly decreased. These results suggest abnormal creatine metabolism in cancer cachexia. To test the hypothesis that creatine supplementation could alleviate the pathological phenotype of cancer cachexia, we performed an *in vivo* study by administering creatine to cachectic mice. We observed obvious therapeutic benefits of creatine on cachectic weight loss in a tumor-bearing mouse model, and this effect was mediated through inhibition of UPS and ALS activation.

Ubiquitination is an important step in protein degradation, and the binding of ubiquitin and the target protein involves the fine mediation of ubiquitin activating enzyme (E1), ubiquitin binding enzyme (E2), and ubiquitin ligase (E3). E3 ubiquitin ligases specific to skeletal muscle include MuRF-1/Trim63, Atrogin-1/MafBx/Fbxo32, Trim32, and Nedd4-1. Among these E3 ubiquitin ligases, MuRF-1 and Atrogin-1 play key roles in skeletal muscle atrophy in malignant tumor masses (Bilodeau et al., 2016). Mouse knockout (KO) experiments demonstrated that these two UPS genes are quite important for muscle atrophy, regulating the degradation of myogenic fibronectin, leading to loss of muscle strength, mass, and function (Gomes et al., 2001; Clarke et al., 2007; Fielitz et al., 2007). In our study, we observed significantly increased expression of skeletal muscle-specific E3 ubiquitin ligases, including MuRF-1 and Atrogin-1, suggesting the activation of the UPS in cachectic skeletal muscle. Creatine supplementation inhibited their expression and blocked UPS activation.

The activation of ALS is also involved in the process of skeletal muscle atrophy (Masiero and Sandri, 2010), and inactivation of the PI3K/AKT pathway contributes to this process. PI3K/Akt plays an important role in myotubular hypertrophy, and activation of its downstream target mTOR can increase skeletal muscle mass and enhance skeletal muscle function (Sakai et al., 2021). A previous study reported that PI3K/Akt also inhibits FoxOs and suppresses the transcription of E3 ubiquitin ligases that regulate UPS-mediated protein degradation. Our results demonstrated that in cancer cachexia, energy deficiency *in vivo* reduced Akt activation, which in turn released its inhibitory effects on UPS and ALS pathway activation, leading to skeletal muscle atrophy and degradation. Therefore, improving energy production in cancer cachexia is a key aspect in studying therapeutic strategies for cancer cachexia.

ATP is the only form of energy utilized by the body, and skeletal muscle contractile exercise requires large amounts of ATP; however, ATP is stored in very small amounts in the body, and stored ATP is quickly depleted (Muccini et al., 2021). Unlike ATP, creatine can be stored in skeletal muscle and functions as a transient ATP pool that can briefly and rapidly provide the body with large amounts of ATP (Owen and Sunram-Lea, 2011). In our study, we detected reduced creatine synthesis in the liver of cancer cachectic mice, accompanied by decreased creatine transport and increased expression of creatine kinase skeletal muscle isoform (CKM) in cachectic muscle. We observed that creatine supplementation decreased CKM expression and increased creatine content but did not influence creatine transport in cachectic skeletal muscle. These findings indicate that oral creatine administration can increase the creatine content in skeletal muscle by increasing hepatic creatine synthesis *in vivo*, thus stimulating ATP generation and alleviating skeletal muscle atrophy in cancer cachexia. Importantly, we also observed that the inhibition of muscle wasting by creatine resulted in improved function, as measured by forelimb grip strength, in mice with cancer cachexia.

As the main site of ATP production, mitochondrial changes in skeletal muscle in cachexia are of interest. Mitochondria play a central role in bioenergetics and metabolism and are often referred to as eukaryotic power stations, where several important processes in cellular respiration occur, such as the Krebs cycle, oxidative phosphorylation, and fatty acid β-oxidation, and oxidative phosphorylation is the most critical energy production pathway (Mollazadeh et al., 2021). Mitochondrial dysfunction includes different features, such as reduced mitochondrial content, altered mitochondrial morphology, reduced electron transport chain complex activity, open mitochondrial permeability transition pores, and increased reactive oxygen species (ROS) formation, and tissues that are more affected by mitochondrial dysfunction are usually those with high energy requirements, such as skeletal muscle and the heart (Boengler et al., 2017). Our study showed that there is dysfunction of skeletal muscle mitochondria in cancer cachexia, including the occurrence of mitochondria, mitochondrial fission/fusion, and abnormal expression of mitochondrial complexes, whereas the number, morphology, and function of mitochondria are impaired, and creatine supplementation corrects this abnormal change, restores mitochondrial morphology and function to some extent, activates Akt, inhibits the UPS and ALP systems, and thereby protects skeletal muscle from degradation.

In addition to the protective effects on skeletal muscle atrophy, we also noticed the protective effects of creatine on adipose tissue. PGC-1α is a coactivator of several transcription factors and a key regulator of mitochondrial biogenesis, energy homeostasis, adaptive thermogenesis and glucose metabolism and has been shown to regulate key components of adaptive thermogenesis, including uncoupling respiration *via* mitochondrial uncoupling protein (UCP-1), which causes white fat browning for heat production (Chaturvedi and Beal, 2013). Our data demonstrate that creatine supplementation significantly reduces the expression levels of PGC-1α and UCP-1, thus inhibiting the process of adipose tissue browning to protect against adipose tissue wasting in cachectic mice. Apart from the influence of cachectic muscle wasting, we also noticed the inhibitory effects of creatine on tumor growth in C26 or LLC tumor-bearing mice. Some previous studies have shown that additional creatine supplementation could increase the risk of cancer metastasis and shorten mouse survival in some tumor-bearing mouse models (Sakkas et al., 2009; Prado et al., 2020; Candow et al., 2022). However, our results did not support this point; in response to creatine treatment, both the tumor weight and cancer metastasis were not significantly altered in either the C26 or LLC tumor-bearing mouse models. We propose that this observation might be due to the different cellular heterogeneity in many types of cancers. However, it is important to note, that our study has some limitations. A major limitation of the present study is the lack of histopathology analysis of the tumor itself. Our study used only BALB/c and C57BL/6 male mice since both sexes may exhibit differences in mitochondrial markers, so more research must be conducted using both sexes. The effect of creatine supplementation on one-carbon metabolism also needs to be investigated in future studies.

In summary, our study showed that creatine supplementation can inhibit the activation of UPS and ALP in skeletal muscle by activating the Akt signaling pathway, increase the creatine content in skeletal muscle, and provide ATP to skeletal muscle. It can also reduce the thermogenic effect of adipose tissue in cachexia by inhibiting the browning of white adipose tissue and significantly improving the atrophic degradation of skeletal muscle and adipose tissue in tumor cachexia, which provides a basis for nutritional intervention in clinical cancer cachexia patients.

## Data Availability

The original contributions presented in the study are included in the article/Supplementary Material, further inquiries can be directed to the corresponding author.

## Supplementary material

Supplementary Figure S1 Creatine treatment alleviated cachexic-associated weight loss and muscle wasting in LLC tumor-bearing mice. **(A)** Representative general images, skeletal muscle images and homologous groin images of the four groups. NC group: normal control mice, cachexia group: C26 tumor-bearing mice, NC+Cr group: Creatine treated normal control mice, cachexia+Cr group: Creatine treated C26 tumor-bearing mice. **(B)** Body weight changes in the four groups (mean ± SD, n = 6). Day 0: Tumor implantation. Day 7: Creatine injection began. Day 15: Terminal day. ****p* < 0.001 denoted the NC group versus the cachexia group, ***p* < 0.01 denoted the cachexia group versus the cachexia+Cr group. **(C)** Tumor removal weight changes in the four groups (mean ± SD, n = 6). ***p* < 0.01 denoted the NC group versus the cachexia group, **p* < 0.05 denoted the cachexia group versus the cachexia+Cr group. **(D)** The lean mass changes in the four groups (mean ± SD, n = 6). **p* < 0.05 denoted the NC group versus the cachexia group, ***p* < 0.01 denoted the cachexia group versus the cachexia+Cr group. **(E)** iWAT mass changes in the four groups (mean ± SD, n = 6). **p* < 0.05 denoted the NC group versus the cachexia group. ***p* < 0.01 denoted the cachexia group versus the cachexia+Cr group. There was no significant difference between the cachexia group and the cachexia+Cr group. **(F)** The tumor weight changes in the cachexia group and the cachexia+Cr group (mean ± SD, n = 6). There was no significant difference between the cachexia group and the cachexia+Cr group. **(G)** The heart weight changes in the four groups (mean ± SD, n = 6). There was no significant difference between the cachexia group and the cachexia+Cr group. **(H)** Daily food consumption in the four groups during the course of the experiment. (mean ± SD, n = 6). There was no significant difference between the cachexia group and the cachexia+Cr group.
