# Supplementary figures and images for: Creatine modulates cellular energy metabolism and protects against cancer cachexia-associated muscle wasting
Source: Front Pharmacol. 2022 Dec 7;13:1086662. doi: 10.3389/fphar.2022.1086662 (PMC9767983; doi:10.3389/fphar.2022.1086662)

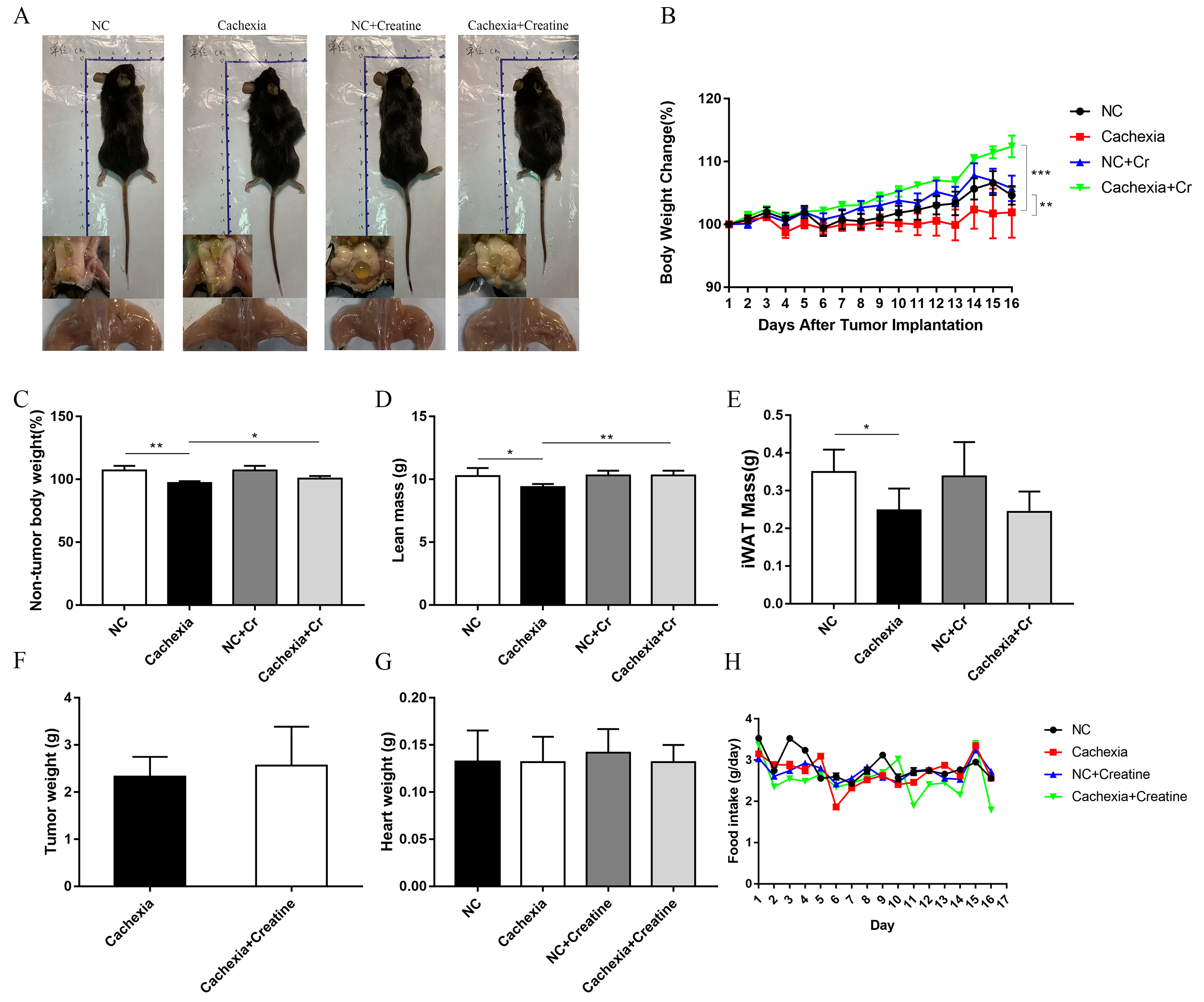

Supplement: Supplementary file 1 [file Presentation1.zip › Supplementary Fig 1/Supplementary Fig 1.tif]
